# Microbial factories for recombinant pharmaceuticals

**DOI:** 10.1186/1475-2859-8-17

**Published:** 2009-03-24

**Authors:** Neus Ferrer-Miralles, Joan Domingo-Espín, José Luis Corchero, Esther Vázquez, Antonio Villaverde

**Affiliations:** 1 Institut de Biotecnologia i de Biomedicina, Universitat Autònoma de Barcelona, 08193 Barcelona, Spain; 2 Department de Genètica i de Microbiologia, Universitat Autònoma de Barcelona, 08193 Barcelona, Spain; 3 CIBER de Bioingeniería, Biomateriales y Nanomedicina (CIBER-BBN), Barcelona, Spain

## Abstract

Most of the hosts used to produce the 151 recombinant pharmaceuticals so far approved for human use by the Food and Drug Administration (FDA) and/or by the European Medicines Agency (EMEA) are microbial cells, either bacteria or yeast. This fact indicates that despite the diverse bottlenecks and obstacles that microbial systems pose to the efficient production of functional mammalian proteins, namely lack or unconventional post-translational modifications, proteolytic instability, poor solubility and activation of cell stress responses, among others, they represent convenient and powerful tools for recombinant protein production. The entering into the market of a progressively increasing number of protein drugs produced in non-microbial systems has not impaired the development of products obtained in microbial cells, proving the robustness of the microbial set of cellular systems (so far *Escherichia coli *and *Saccharomyces cerevisae*) developed for protein drug production. We summarize here the nature, properties and applications of all those pharmaceuticals and the relevant features of the current and potential producing hosts, in a comparative way.

## Introduction

**Figure 1:**
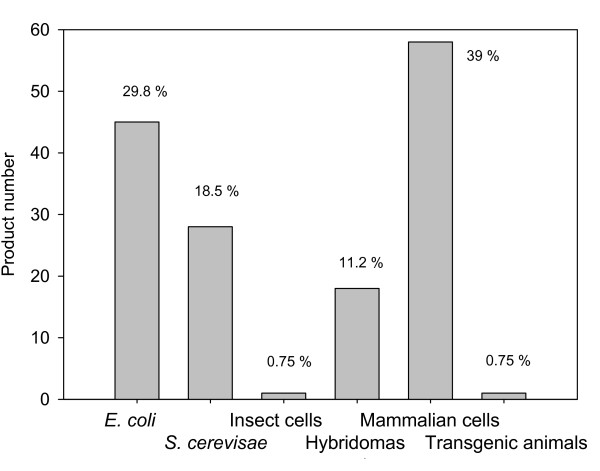
**Number (and percentage values siding the bars) of recombinant proteins approved as biopharmaceuticals in different production systems**. Data has been adapted from Table 1 in [3]. Exubera, an inhalated recombinant human insulin produced in *E. coli *has been omitted since Pfizer stopped its marketing in January 2008. Two recently FDA approved products Xyntha and Recothrom produced both in CHO cells have also been added.

Proteins are catalysers of metabolic reactions, structural components of biological assemblies, and responsible for inter and intracellular interactions and cell signalling events that are critical for life. Therefore, deficiencies in the production of specific polypeptides or the synthesis of mutant, non-functional versions of biologically relevant protein usually derive in pathologies that can range from mild to severe. In humans, such diseases can be treated by the clinical administration of the missing protein from external sources, to reach ordinary concentrations at systemic or tissular levels [1]. Therefore, many human proteins have an important pharmaceutical value but they are difficult to obtain from their natural sources. Recombinant DNA (rDNA) technologies, developed in the late 70's using the bacterium *Escherichia coli *as a biological framework, offer a very potent set of technical platforms for the controlled and scalable production of polypeptides of interest by relatively inexpensive procedures. This can be done in convenient microbial cells such as bacteria and yeasts, whose cultivation can be accomplished by relatively simple procedures and instrumentation. In early 80's, the FDA approved the clinical use of recombinant human insulin from recombinant *E. coli *(Humulin-US/Humuline-EU) for the treatment of diabetes [2], being the first recombinant pharmaceutical to enter the market. The versatility and scaling-up possibilities of the recombinant protein production opened up new commercial opportunities for pharmaceutical companies. Since the approval of recombinant insulin, other recombinant DNA drugs have been marketed in parallel with the development and improvement of several heterologous protein production systems. This has generated specific strains of many microbial species adapted to protein production, and has allowed the progressive incorporation of yeasts and eukaryotic systems for this purpose. Among the 151 protein-based recombinant pharmaceuticals licensed up to January 2009 by the FDA and EMEA, 45 (29.8%) are obtained in *Escherichia coli*, 28 (18.5%) in *Saccharomyces cerevisiae*, 17 (11.2%) in hybridoma cells, 1 in transgenic goat milk, 1 in insect cells and 59 (39%) in mammalian cells (Figure 1) [3]. In the next sections, the key properties of these expression systems will be analyzed regarding both the biological convenience and final quality of the products. Alternative promising protein production systems such as filamentous fungi, cold-adapted bacteria and alternative yeast species among others are under continuous development but only few biopharmaceutical products from them have been marketed. Relevant properties of such promising systems and their potential as producers of therapeutic proteins have been extensively reviewed elsewhere [4-12].

### Escherichia coli

The enterobacterium *E. coli *is the first-choice microorganism for the production of recombinant proteins, and widely used for primarily cloning, genetic modification and small-scale production for research purposes. This is not surprising as the historical development of microbial physiology and molecular genetics was mainly based on this species, what has resulted in a steady accumulation and worldwide use of both information and molecular tools (such as engineered phages, plasmids and gene expression cassettes). However, several obstacles to the production of quality proteins limit its application as a factory for recombinant pharmaceuticals. Recombinant proteins obtained in *E. coli *lack the post-translational modifications (PTMs) which are present in most of eukaryotic proteins [13]. Glycosylation is the most common PTM [14] but many others, such as disulfide bond formation, phosphorylation and proteolytic processing might be essential for biological activity. PTMs play a crucial role in protein folding, processing, stability, final biological activity, tissue targeting, serum half-life and immunogenicity of the protein; therefore PMT deficient version might be insoluble, unstable or inactive. Interestingly, it is possible to attach or bind synthetic PTMs in the case of pegylated products [15] such as human growth hormone, granulocyte colony stimulating factor, interferons alfa-2a and alfa-2b, which renders versions of the protein in serum more stable than the naked product. Also, the N-linked glycosylation system of *Campylobacter jejuni *has been successfully transferred to *E. coli*, making this approach a promising possibility for the production of glycosilated proteins in this species [16]. Furthermore, through genetic engineering of the underlying DNA, the amino acid sequence of the protein can be changed to alter its ADME (absorption, distribution, metabolism, and excretion) properties, as it has been observed for insulin (Table 1) [17].

**Table 1:** Recombinant insulins approved for human use.

**INN^1^**	**Trade name**	**Production system**	**Modifications from natural**	**PK^2^**
Insulin human	Humulin Insuman Exubera^3^	*E. coli*	None	Short-acting insulin
Insulin human	Novolin	*S. cerevisiae*	None	Short-acting insulin

Insulin lispro	Humalog	*E. coli*	PB28K and KB29P	Rapid-acting insulin analogue
Insulin glulisine	Apidra	*E. coli*	NB3K and KB29E	Rapid-acting insulin analogue
Insulin aspart	Novorapid	*S. cerevisiae*	DB28P	Rapid-acting insulin analogue

Insulin glargin	Lantus	*E. coli*	NA21G and 2 additional R in B chain	Long-acting insulin analogue
Insulin detemir	Levemir	*S. cerevisiae*	TB30del and myristic fatty acid attached to KB29 by acylation	Long-acting insulin analogue

Insulin is a polypeptide of 51 amino acid, 30 of which constitute A chain, and 21 of which comprise B chain. The two chains are linked by a disulfide bond. Mutations in amino acid sequences are noted for each of the chains.

^1^INN: International Nonproprietary Names. ^2^PK:PharmacoKinetics. ^3^Exubera: Rapid-actin insulin using inhalation route [17], was discontinued in 2008 by the manufacturer

On the other hand, the frequencies with which the different codons appear in *E. coli *genes are different from those occurring in human genes, and this is directly related to the abundance of specific tRNAs. Therefore, genes that contain codons rare for *E. coli *may be inefficiently expressed by this organism and cause premature termination of protein synthesis or amino acid misincorporation, thus reducing the yield of expected protein versions [18]. This problem can be solved either by site-directed replacement of rare codons in the target gene by codons that are more frequently used in *E. coli*, or, alternatively, by the co-expression of the rare tRNAs (*E. coli *strains BL21 codon plus and Rosetta were designed for this purpose). In addition, initial methionine removal depends on the side chain of the penultimate amino acid of N-terminal in final recombinant proteins produced in *E. coli *although it can be efficiently removed using recombinant methionine aminopeptidase [19]. Some mutant *E. coli *strains have been developed to promote disulfide bond formation (AD494, Origami, Rosetta-gami) and/or with reduced protease activity (BL21). As an additional technical obstacle, proteins larger than 60 kDa are inefficiently obtained in soluble forms in *E. coli *[20].

As it has been well documented, bacteria overproducing either eukaryotic or prokaryotic recombinant proteins are subjected to different stresses (essentially metabolic and conformational) [21]. Under this situation, protein processing associated to cell stress responses might render non useless products, mainly because of lack of solubility, and many protein species deposit in high amounts as protein aggregates known as inclusion bodies (IBs) [22-25]. By adjusting media composition, growth temperature, inducer concentration, promoter strength and plasmid copy number, variable amounts of the target protein can be forced to appear in the soluble form [26,27], although unfortunately, many eukaryotic proteins are exclusively found trapped in IBs and seem to be resistant to process-based solubility enhancement. While IBs formed by enzymes can be efficient catalysers in enzymatic reactions [28-32], pharmaceutical proteins need, in contrast, to be dispersed as soluble entities to reach their targets at therapeutic doses. IBs essentially contain the recombinant protein in variable proportions (from 60 to more than 90%) and some contaminants as chaperones, DNA, RNA and lipids [33]. Although stored protein can be released from IBs using denaturing conditions, in vitro refolding processes are not as effective as expected [34] and other expression systems should be tried. In some cases, recombinant proteins have been successfully purified from IBs as for example Betaferon [35] and insulin [36]. However, for non integral membrane proteins, cytosolic and/or soluble protein domains, the probability of success is reasonably high and *E. coli *should be then considered as a promising expression system [37].

**Figure 2:**
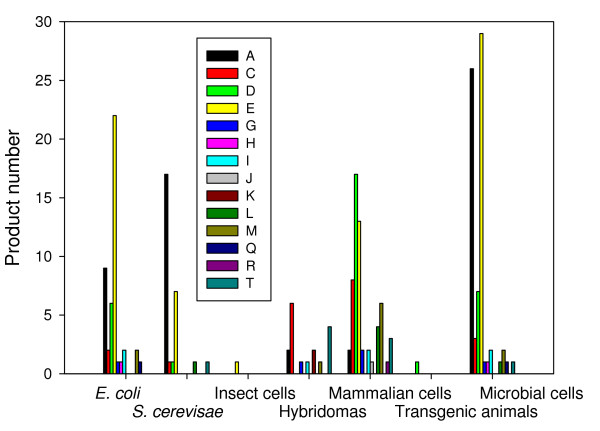
**Number of recombinant biopharmaceuticals in different production systems, grouped by WHO therapeutic indications (see the legend of Additional file for nomenclature)**. Products from *E. coli *and *S. cerevisae *are also presented together under the category of microbial cells.

In summary, around 10% of full-length eukaryotic proteins tested in this system have been successfully produced in soluble form in *E. coli *[38]. Approved therapeutic protein-based products from *E. coli *include hormones (human insulin and insulin analogues, calcitonin, parathyroid hormone, human growth hormone, glucagons, somatropin and insulin growth factor 1), interferons (alfa-1, alfa 2a, alfa-2b and gamma-1b), interleukins 11 and 2, light and heavy chains raised against vascular endothelial growth factor-A, tumor necrosis factor alpha, cholera B subunit protein, B-type natriuretic peptide, granulocyte colony stimulating factor and plasminogen activator (Additional file 1). Noteworthy, most of the recombinant pharmaceuticals produced in *E. coli *are addressed for the treatment of infectious diseases or endocrine, nutritional and metabolic disorder disease groups (Figure 2).

### Saccharomyces cerevisiae

Production in yeast is usually approached when the target protein is not produced in a soluble form in the prokaryotic system or a specific PTM, essential for its biological activity, cannot be produced artificially on the purified product [13]. Yeasts are as cost effective, fast and technically feasible as bacteria and high density cell cultures can also be reached in bioreactors. Even more, mutant strains that produce high amounts of heterologous protein are already available. Even though yeasts are able to perform many PTMs as O-linked glycosylation, phosphorylation, acetylation and acylation, the main pitfall of this expression system is related to N-linked glycosylation patterns which differ from higher eukaryotes, in which sugar side chains of high mannose content affect the serum half-life and immunogenicity of the final product. Although less studied than in bacteria, the production of recombinant proteins also triggers conformational stress responses and produced proteins fail sometimes to reach their native conformation. Recent insights about conformational stress, and in general, to cell responses to protein production in recombinant yeasts have been extensively reviewed elsewhere [21,39,40].

The approved protein products produced in yeast are obtained exclusively in *Saccharomyces cerevisiae *[4] and correspond to hormones (insulin, insulin analogues, non glycosylated human growth hormone somatotropin, glucagon), vaccines (hepatitis B virus surface antigen -in the formulation of 15 out of the 28 yeast derived products-) and virus-like particles (VLPs) of the major capsid protein L1 of human papillomavirus type 6, 11, 16 and 18, urate oxidase from *Aspergillus flavus*, granulocyte-macrophage colony stimulating factor, albumin, hirudin of *Hirudo medicinalis *and human platelets derived growth factor. As in the case of *E. coli*, most of the recombinant pharmaceuticals from yeast are addressed to either infectious diseases or endocrine, nutritional and metabolic disorders (Figure 2), being these therapeutic areas the most covered by microbial products. Interestingly, several yeast species other than *S. cerevisiae *are being explored as sources of biopharmaceuticals and other proteins of biomedical interest [21,41]. In addition, current metabolic engineering approaches [42] and optimization of process procedures [43,44] are dramatically expanding the potential of yeast species for improved production of recombinant proteins.

### Insect cell lines

Cultured insect cells are used as hosts for recombinant baculovirus infections. The production of a recombinant viral vector for gene expression is time-consuming, the cell growth is slow when compared with former expression systems, the cost of growth medium is high and each protein batch preparation has to be obtained from fresh cells since viral infection is lethal. PTMs are also an important limitation of this expression system because of the simple non-syalated N-linked glycosylation which is translated in a rapid clearance from human sera [45]. Although genetic engineering has been used to select transgenic insect cell lines (MIMIC™ from Invitrogen and SfSWT-3) expressing galactosyltransferase, N-acetylglucosaminyltransferases, syalic acid synthases and syaliltransferases genes [46-48] to obtain humanized complex N-linked glycosylation protein patterns, there are still unwanted toxicological issues that need to be overcome.

There is only one approved biopharmaceutical product containing recombinant proteins from infected insect cell line Hi Five, Cervarix, consisting on recombinant papillomavirus C-terminal truncated major capsid protein L1 types 16 and 18. Nonetheless, this expression system has been extensively used in structural studies since correctly folded eukaryotic proteins can be obtained in a secreted form in serum free media which enormously simplifies protein capture in purification protocols.

### Hybridoma cell lines

Hybridomas are fusion cells of murine origin (B-cells and myeloma tumour cells) that are able to express specific monoclonal antibodies against a determined antigen, thus possessing therapeutic potential [49]. Clone selection may account for the progressive enrichment of cells displaying a glycosylation profile with reduced potency and undesirable immunogenic reaction since the human immune system recognizes mouse antibodies as foreign.

Genetic engineering has been applied to obtain humanized monoclonal antibodies using either recombinant mammalian cells producing chimeric antibodies or genetically modified mice to produce human-like antibodies [49]. One such product, Remicade, which binds tumour necrosis factor-alpha, is a pharmaceutical blockbuster used in the treatment of Crohn's disease.

### Hamster cell lines

Most of the therapeutic proteins approved so far have been obtained using transgenic hamster cell lines, namely 49 in chinese hamster ovary cells (CHO) and 1 in baby hamster kidney cells (BHK) (Additional file 1). The main advantage of this expression system is that cells can be adapted to grow in suspension in serum free media (SFM), protein-free and chemically defined media [50]. This fact increases the biosafety of final products reducing risk of introducing prions of bovine spongiform encephalopathy (BSE) from bovine serum albumin and of infectious variant Creutzfeldt-Jakob Disease (vCJD) from human serum albumin. In addition, recombinant products can be secreted into the chemical defined media, which simplifies both upstream and downstream purification process [51]. PTMs in this expression system are almost the same as in human cell lines, although some concerns about comparability in the glycosylation pattern have arisen when comparing different batches of the same manufacturer product and biosimilars [52]. Further development of chemically defined media and fine description of growth conditions would help to overcome this issue.

### Human cell lines

In the recent years, three therapeutic proteins produced in human cell lines have been approved, namely Dynepo-erithropoietin, Elaprase-irudonate-2-sulfatase and Replagal-alfa-galactosidase A. These products are fully glycosylated human proteins, so this expression system should be addressed when heavily glycosylation is needed. In general, recombinant biopharmaceuticals obtained from mammalian cells cover a wider spectrum of pathological conditions than those obtained from microbes, and the distribution of applications is less biased than when observing products from *E. coli *or *S. cerevisae *(Figure 2).

### Transgenic animals

Transgenic animals (avian and mammals), have been successfully used for the production of recombinant proteins secreted into egg white and milk respectively. Protein production using transgenic farm animals supposes a great biotechnological challenge in terms of safety concerns such as transmission of infectious diseases (including viral and prion infections) or adverse allergenic, immunogenic and autoimmune responses. In 2006, ATryn was the first and so far single approved rDNA biopharmaceutical using transgenic animals and validated manufacturer technology platform. It contains human antithrombin (432 amino acids) with 15% glycosylated moieties and is secreted into the milk of transgenic goats. Another product obtained from the milk of transgenic rabbits (Rhucin) has been recently denied for its approval by the EMEA although more tests of repeated treatment are underway to try again its approval. Despite such limited progress, if pharmacovigilance after patient treatment does not reveal any adverse side effects, we might envisage, in the next years, an increase in the approval rate of recombinant protein products from transgenic animal origin.

### Alternative, non microbial systems for forthcoming products

As previously discussed, recombinant DNA biopharmaceuticals obtained from bacterial, yeast or mammalian cell culture bioreactors are quite effective as therapeutic agents although production costs are relatively high. One way to address the economic-cost benefit hurdle is through the use of transgenic organisms to manufacture biopharmaceuticals. Biopharming would dramatically reduce the cost of recombinant therapeutic proteins not only in the initial construction of production facilities but also the scale-up process and the final recombinant protein yield. Nonetheless, the fact that regulatory guidelines are being developed as the same time that the establishment of protein production processes is creating uncertainty within biotechnological companies to fulfil drug administration requirements.

Transgenic plants have been used as recombinant protein producers for research and diagnostic uses due to the advantageous low cost of cultivation, high mass production, flexible scale-up, lack of human pathogens and addition of eukaryotic PTMs. The first recombinant protein product obtained from transgenic tobacco was human growth hormone [53] and since then, many other products have been obtained (including antibodies, the surface antigen of the Hepatitis-B-Virus, industrial enzymes and milk proteins). Again, the main disadvantage is related to the plant specific PMTs introduced in recombinant proteins which produce adverse immune responses. Moreover, the possibility to spread the proteins in open fields and the negative public perception of the transgenic plants precludes the use of plants as an attractive expression system of therapeutic proteins.

### Host comparative trends in rDNA biopharmaceutical approval

**Figure 3:**
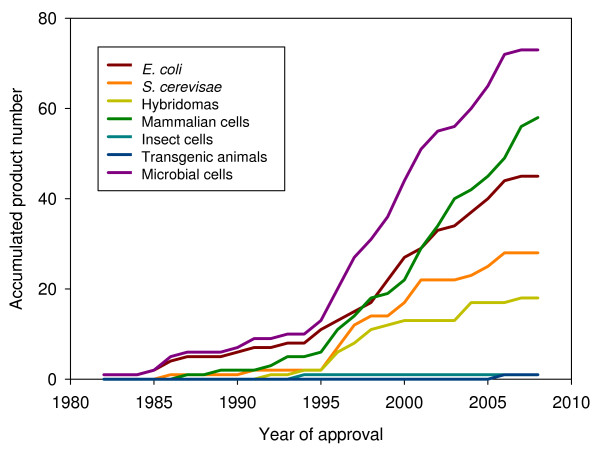
**Accumulated number of recombinant biopharmaceuticals obtained in different production systems, in front of year of their first time approval (either in US or EU)**. Products from *E. coli *and *S. cerevisae *are presented together under the category of microbial cells.

As mentioned above, human insulin produced in *E. coli *was the first rDNA pharmaceutical approved for use, which was followed by a progressively increasing number of other protein drugs from bacteria and yeast (Figure 3). Since 1995, the progression of products of mammalian origin was noticeable and extremely regular, and quantitatively comparable to that of microbial products. Importantly, the incorporation of mammalian cells as factories for rDNA pharmaceuticals has neither represented an excluding alternative to microbial hosts nor resulted in a decrease in the approval rate of microbial products (Figure 3). This is probably due to the extremely different biologically and technologically backgrounds associated to protein production, the good quality of microbial products and the high costs associated to mammalian cell production. In addition, this fact indicates the potential of microbial cells in biopharmaceutical industry despite the limited PTM performance of their products and other bottlenecks as discussed above. Also, microbial cell factory products cover a spectrum of products and application fields that do not necessarily match those addressed by mammalian cell factories (Figure 2).

Interestingly, a plateau in the rate of rDNA drug approval during the last 2–3 years is becoming perceivable, irrespective of the production system (Figure 3). Although it might be observed as a transient event, this fact seems instead to indicate that the current production systems could be near to the exhaustion regarding their ability to hold the production of complex proteins, protein complexes or the so-called difficult-to-express proteins. Desirably, recent insights about system's biology of recombinant cells and hosts, and specially, arising novel concepts on recombinant protein quality [54-56] and host stress responses [21] would enlarge the possibilities for metabolic and process engineering aiming to the economically feasible production of new, more complex drugs. Indeed, pushed by fast advances in molecular medicine the pharmaceutical industry is urgently demanding improved production systems and novel and cheaper drugs.

## Conclusions and future prospects

Overcoming the biological and methodological obstacles posed by cell factories to the production or rDNA pharmaceuticals is a main challenge in the further development of protein-based molecular medicine. Recombinant DNA technologies might have exhausted conventional cell factories and new production systems need to be deeply explored and incorporated into the production pipeline. On the other hand, a more profound comprehension of host cell physiology and stress responses to protein production would necessary offer improved tools (either at genetic, metabolic or system levels) to favour high yield and high quality protein production. Apart from the expected incorporation of unusual mammalian hosts such as transgenic animals or plants, microbial cells appear as extremely robust and convenient hosts, and gaining knowledge about the biological aspects of protein production would hopefully enhance the performance of such hosts beyond the current apparent limitations. In this regard, not only commonly used bacteria and yeasts but unconventional strains or species are observed as promising cell factories for forthcoming recombinant drugs. Their incorporation into productive processes for human pharmaceuticals would hopefully push the trend of marketed products and fulfil the increasing demands of the pharmacological industry.

## Abbreviations

(ADME): absorption, distribution, metabolism, and excretion; (BHK): baby hamster kidney cells; (CHO): chinese hamster ovary cells; (EMEA): European Medicines Agency; (FDA): Food and Drug Administration; (IBs): inclusion bodies; (PTMs): post-translational modifications; (rDNA): Recombinant DNA; (VLPs): virus-like particles

## Competing interests

The authors declare that they have no competing interests.

## Authors' contributions

All authors read and approved the manuscript's content.

## Supplementary Material

Additional file 1 Recombinant drugs approved for use, grouped by producing host types. Click here for file
