# Supplementary material for: Microbial factories for recombinant pharmaceuticals
Source: Microb Cell Fact. 2009 Mar 24;8:17. doi: 10.1186/1475-2859-8-17 (PMC2669800; doi:10.1186/1475-2859-8-17)
Supplement: Additional file 1 — Recombinant drugs approved for use, grouped by producing host types. [file 1475-2859-8-17-S1.doc]

**Table**: Recombinant drugs approved for use, grouped by producing host types.

| **Product name and nature** | **WHO**  **indication ^a^** | **Approval date and place** |
| --- | --- | --- |
| **Host: *E. coli*** | | |
| Dukoral (Oral cholera vaccine) | A | 2004 EU, US |
| Pegasys (Peginterferon alfa-2a) |  | 2002 US |
| PegIntron (Peginterferon alfa-2b) |  | 2000 EU 2001 US |
| Infergen (Interferon alfacon-1) |  | 1997 US 1999 EU |
| Rebetron (Interferon alfa-2b) |  | 1999 US |
| Roferon A (Interferon alfa-2ª) |  | 1986 US |
| Viraferon (Interferon alfa-2b) |  | 2000 EU |
| ViraferonPeg (Peginterferon alfa-2b) |  | 2000 EU |
| Intron A (Interferon alfa-2b) | A, C | 1986 US 2000 EU |
| Beromun (Tasonermin) | C | 1999 EU |
| Actimmune (Interferon gamma-1b) | D | 1990 US |
| IPLEX (Mecasermin rinfabate recombinant) |  | 2005 US |
| Kepivance (Palifermin) |  | 2004 EU 2005 US |
| Neulasta (Pegfilgrastim) |  | 2002 US 2002 EU |
| Neumega (Oprelvekin) |  | 1997 US |
| Neupogen (Filgrastim) |  | 1991 US |
| Humalog (Insulin lispro) | E | 1996 US, EU |
| Humatrope (Somatotropin) |  | 1987 US |
| Humulin (Human insulin) |  | 1982 US |
| Insuman (Human insulin) |  | 1998 EU |
| Lantus (Insulin glargine) |  | 2000 EU, US |
| Fortical (Salmon calcitpnin) |  | 2005 US |
| Apidra (Insulin glulisine) |  | 2004 EU, US |
| Exubera (Human insulin) |  | 2006 EU |
| Forcaltonin (Salmon calcitonin) |  | 1999 EU |
| Forsteo (Teriparatide) |  | 2003 EU |
| Forsteo/Forteo (Teriparatide) |  | 2002 US 2003 EU |
| Genotropin (Somatotropin) |  | 1995 US |
| Glucagon |  | 1998 US |
| Increlex (Mecasermin) |  | 2005 US 2007 EU |
| Insulin human Winthrop (Insulin human) |  | 2007 EU |
| Norditropin (Somatropin) |  | 1995 US |
| Nutropin (Somatropin) |  | 1999 US |
| NutropinAQ (Somatropin) |  | 2001 EU 1995 US |
| Optisulin (Insulin glargine) |  | 2000 EU |
| Preotact (Human parathyroid hormone) |  | 2006 EU |
| Protropin (Somatrem) |  | 1985 US |
| Somavert (Pegvisomant) |  | 2002 EU 2003 US |
| Betaferon/Betaseron (Interferon beta-1b) | G | 1993 US 1995 EU |
| Lucentis (Ranibizumab) | H | 2006 US 2007 EU |
| Natrecor (Nesiritide) | I | 2001 US |
| Rapilysin/Retavas e (Reteplase) |  | 1996 EU, US |
| Ontak (Denileukin diftitox) | M | 1999 US |
| Kineret (Anakinra) |  | 2001 US 2002 EU |
| Omnitrope (Somatropin) | Q | 2006 EU |
|  |  |  |
| **Host: *S .cerevisiae*** | | |
| Ambirix (Hepatitis A inactivated, hepatitis B antigen (rDNA)) | A | 2002 US |
| Comvax (Meningococcal protein conjugate) |  | 1996 US |
| Engerix-B (Hepatitis B vaccine) |  | 1998 US |
| Fendrix (Hepatitis B vaccine) |  | 2005 EU |
| Gardasil/Silgard (Human papilomavirus vaccine [type 6,11,16,18]) |  | 2006 EU, US |
| HBVAXPRO (Hepatitis B vaccine) |  | 2001 EU |
| Hexavac (Hexavalent vaccine, hepatitis B (rDNA)) |  | 2000 EU |
| INFANRIX HepB (Tetravalent vaccine, hepatitis B (rDNA)) |  | 1997 EU |
| Infanrix Hexa (Hexavalent vaccine, hepatitis B (rDNA)) |  | 2000 EU |
| Infanrix Pent/Pediatrix(Pentavalent vaccine, hepatitis B (rDNA)) |  | 2000 EU 2002 US |
| M-M-RVAXPRO (Measles, mumps and rubella vaccine) |  | 2006 EU |
| Procomvax/comvax (Mixed vaccines) |  | 1999 EU 1996 US |
| Quintanrix (Mixed vaccines) |  | 2005 EU |
| Recombivax (Hepatitis B vaccine) |  | 1986, 1999, 2000 US |
| Tritanrix HepB (Mixed vaccines) |  | 1996 EU |
| Twinrix Adult (Mixed vaccines) |  | 1996 EU 2001 US |
| Twinrix Paediatric (Mixed vaccines) |  | 1997 EU |
| Leukine (Sargramostim) | C | 1991 US |
| Refludan (Lerpirudin) | D | 1997 EU 1998 US |
| Fasturect (Rasburicase) | E | 2001 EU |
| Fasturetec/Elitek (Rasburicase) |  | 2001 EU 2002 US |
| GlucaGen (Glucagon) |  | 1998 US |
| Levemir (Insulin detemir) |  | 2004 EU 2005 US |
| Novolin/Actrapid/ Insulatard/Mixtard /Monotrad/Ultrata rd/Velosulin (Human insulin) |  | 2002 EU 2001 US |
| NovoRapid Novolog/NovoMi x 30/NovoLog Mix 70/30 (Insulin) |  | 1999 EU 2000 US |
| Valtropin (Somatropin) |  | 2006 EU |
| Regranex (Becaplermin) | L | 1997 US 1999 EU |
| Revasc (Desirudin) | T | 1997 EU |
|  |  |  |
| **Host: Insect cells** | | |
| Cervarix (human papillomavirus vaccine) | A | 2007 EU |
|  |  |  |
| **Host: Hybridomas** | | |
| Mylotarg (Gemtuzumab) | A | 2000 US |
| Synagis (Palivizumab) |  | 1998 US 1999 EU |
| CEA-Scan (Arcitumomap) | C | 1996 EU, US |
| Erbitux (Cetuximab) |  | 2004 US, EU |
| HumaSPECT (Votumumab) |  | 1998 EU |
| OncoScint CR/OV (Satumomab) |  | 1992 EU |
| ProstaScint (Capromab pendetide) |  | 1996 US |
| Verluma (Nofetumomab) |  | 1996 US |
| Tysabri (Natalizumab) | G | 2004 US 2006 EU |
| ReoPro (Abciximab) | I | 1994 US |
| NeutroSpec (Fanolesomab) | K | 2004 US, EU |
| leukoScan (Sulesomab) | M | 1997 EU |
| Orthoclone OKT-3 (Murumonab-Cd3) | T | 1996 US |
| Simulect (Basiliximab) |  | 1998 EU 2001 US |
| Soliris (Eculizumab) |  | 2007 EU |
| Zenapax (Daclizumab) |  | 1997 US 1999 EU |
|  |  |  |
| **Host: Mammalian cells** | | |
| Dynepo (Epoetin delta) | A | 2002 EU |
| Xigris (Drotrecogin alfa) |  | 2001 US 2002 EU |
| Avastin (Bevacizumab) | C | 2004 US, EU |
| Erbitux (Cetuximab) |  | 2004 EU, US |
| Herceptin (Trastuzumab) |  | 1998 US 2000 EU |
| MabCampath/Campath (Alemtuzumab) |  | 2001 EU, US |
| MabThera/Rituxan (Rituximab) |  | 1997 US 1998 EU |
| Thyrogen (Thyrotropin alfa) |  | 1998 US 2000 EU |
| Vectibix (Panitumumab) |  | 2006 US |
| Zevalin (Ibritumomab tiuxetan) |  | 2002 US 2004 EU |
| Advata (Octocog alfa) | D | 2004 EU, US |
| Abseamed (Erythropoietin alfa) |  | 2007 US |
| BeneFIX (Nonacog alfa) |  | 1997 US, EU |
| Binocrit (Rh erythropoietin alfa) |  | 2007 EU |
| Bioclate (Antihemophilic factor VIII) |  | 1993 US |
| Epoetin alfa Hexal (Rh erythropoietin alfa) |  | 2007 EU |
| Epogen/Procrit (Epoetin) |  | 1989 US 1990 EU |
| Helixate NexGen,Helixate FS, Kogenate FS (Octocog alfa) |  | 2000 EU 2000 US |
| Mircera (Methoxy polyethylene glycol-epotein beta) |  | 2007 EU |
| NeoRecormon (Epoetin beta) |  | 1997 EU |
| Nespo/Aranesp (Darbepoetin alfa) |  | 2001 EU 2001 US |
| Novoseven (Eptacog alfa) |  | 1996 EU 1999 US |
| Recombinate (Antihaemophilic factor) |  | 1992 US |
| Recothrom (Thrombin) |  | 2008 US |
| ReFacto (Morotcocog alfa) |  | 1999 EU 2000 US |
| Soliris (Eculizumab) |  | 2007 US |
| Xyntha (Morotcocog alfa) |  | 2008 US |
| Aldurazyme (Laronidase) | E | 2003 EU, US |
| Cerezyme (Imiglucerase) |  | 1994 US 1997 EU |
| Elaprase (Idursulfase) |  | 2006 US 2007 EU |
| Fabrazyme (Agalsidase beta) |  | 2001 EU 2003 US |
| Gonal-F (Follitropin alfa) |  | 1995 EU 1997 US |
| Luveris (Lutropin alfa) |  | 2000 EU 2004 US |
| Myozyme (Alglucosidase alfa) |  | 2006 EU, US |
| Naglazyme (Galsulfase) |  | 2005 US 2006 EU |
| Ovitrelle/Ovidrel (Choriogonadotropin alfa) |  | 2001 EU 2001 US |
| Pergoveris (Follitropinalfa/ lutropinalfa) |  | 2007 EU |
| Pulmozyme (Dornase alfa) |  | 1993 US |
| Puregon/Follistim (Follitropin beta) |  | 1996 EU 1997 US |
| Replagal (Agalsidase alfa) |  | 2001 EU |
| Saizen (Somatropin) |  | 1996 US |
| Avonex (Interferon beta-1a) | G | 1996 US 1997 EU |
| Rebif (Interferon beta-1a) |  | 1998 EU 2002 US |
| Activase (Alteplase) | I | 1987 US |
| Metalyse/TNKase (Tenecteplase) |  | 2000 US 2001 EU |
| Xolair (Omalizumab) | J | 2003 US 2005 EU |
| Remicade (Inflixmab) | K | 1999 US 2000 EU |
| Bexxar (Tositumomab) | L | 2003 US |
| Amevive (Alefacept) |  | 2003 US |
| Hylenex (Hyaluronidase human) |  | 2005 US |
| Raptiva (Efalizumab) |  | 2004 EU 2003 US |
| Enbrel (Etanercept) | M | 1998 US 2000 EU |
| InFUSE-InductOs Bone Graf (BMP-2; dibotermin alfa) |  | 2002 US 2007 EU |
| Humira (Adalimumab, human monoclonal antibody) |  | 2002 US 2003 EU |
| Humira (Adalimumab, monoclonal antibody) |  | 2003 EU |
| Osigraft/OP-1 Implant (Eptotermin alfa) |  | 2001 EU 2001 US |
| Serostim (Somatropin) | R | 1996 US |
| InductOs (Dibotermin alfa) | T | 2002 EU |
| Orencia (Abatacept) |  | 2005 US 2007 EU |
| Raptiva (Efalizumab) |  | 2004 EU 2003 US |
|  |  |  |
| **Host: Transgenic animals:** | | |
| ATryn (Antithrombin alfa) | D | 2006 EU |

^a^ WHO indications are as follows (the whole list is available at the URL http://www.who.int/classifications/apps/icd/icd10online/)

A Infectious diseases (Protection or treatment)

C Neoplasms (Imaging or treatment)

D Diseases of the blood and disorders involving the immune mechanism.

E Endocrine, nutritional and metabolic diseases

G Diseases of the nervous system

H Diseases of the eye and the ear

I Diseases of the circulatory system

J Diseases of the respiratory system

K Diseases of the digestive system

L Diseases of the skin and subcutaneous tissue

M Diseases of the musculoskeletal system and connective tissue

N Diseases of the genitourinary system

Q Congenital malformations and chromosomal abnormalities

R Symptoms and signs not elsewhere classified: Pain, cachexia.

T Injuries and surgical complications: Organ rejection.

Y External causes of morbidity and mortality

Z Factors influencing health status and contact with health services
